# Detection of Single Steel Strand Distribution in Grouting Duct Based on Capacitive Sensing Technique †

**DOI:** 10.3390/s19112564

**Published:** 2019-06-05

**Authors:** Nan Li, Mingchen Cao, Hangben Du, Cunfu He, Bin Wu

**Affiliations:** 1 School of Automation, Northwestern Polytechnical University, Xi’an 710071, China; xiaobendu@mail.nwpu.edu.cn; 2 State Key Laboratory for Manufacturing Systems Engineering, Xi’an Jiaotong University, Xi’an 710049, China; mingchen.cao@stu.xjtu.edu.cn; 3 NDT&E Research Centre, Beijing University of Technology, Beijing 100124, China; hecunfu@bjut.edu.cn (C.H.); wb@bjut.edu.cn (B.W.)

**Keywords:** grouting duct, capacitive sensor, cross section distribution detecting, measurement

## Abstract

Grouting ducts (containing steel strands) are widely used to increase the structural strengths of infrastructures. The determination of the steel strand’s integrity inside of ducts and the grouting quality are important for a strength evaluation of the structure. In this study, a capacitive sensing technique was applied to identify the cross-sectional distribution of the steel strands. The distribution was expressed in polar coordinates in an external post-tensioned pre-stressed duct model. An improved capacitive sensor structure was designed, which consisted of four electrodes, and different electrode-pairs were used to determine various locations’ information of the steel strands. Two rounds of measurements were conducted using the designed sensor to detect the angle (*θ*) and center distance (*r*) of the steel strand in the duct. The simulated and experimental results are presented and analyzed. In general, it is difficult to locate the angle of a steel strand directly from first-round capacitance measurements by analyzing the experimental results. Our method based on Q-factor analysis was presented for the position detection of a steel bar in an external post-tensioned pre-stressed duct. The center distance of the steel bar could be identified by second-round capacitance measurements. The processed results verified the effectiveness of the proposed capacitive sensor structure. Thus, the capacitive sensing technique exhibited potential for steel strand cross-section distribution detection in external post-tensioned pre-stressed ducts.

## 1. Introduction

**Figure 1:**
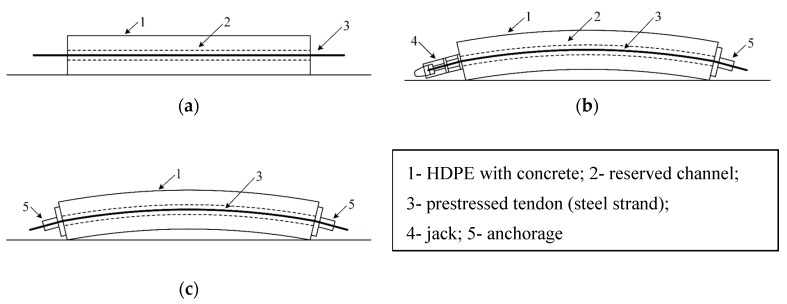
Method of production for external post-tensioned pre-stressed concrete. (**a**) Modeling concrete component; (**b**) pulling steel bar; (**c**) anchoring and grouting.

External post-tensioned pre-stressed technology has been extensively used in the construction of bridges and tunnels. This technology introduces many advantages, including lower component weights, reductions in the vertical shear forces and main tensile stresses of concrete beams, simple structures, and component installation ease. Martin et al. briefly introduced the structures of post-tensioned pre-stressed ducts [1]. In general, pre-stressed steel strands are placed in a HDPE (high-density polyethylene) tube, and high-pressure cement is filled for grouting purposes to prevent corrosion of the steel strand and increase the strength of the structure. The typical method of production for external post-tensioned pre-stressed concrete is illustrated in Figure 1. In the grouting process, an appropriate amount of water-reducing agent should be added to promote cement coagulation. However, in practical grouting operations, the proportion of the water-reducing agent and the grouting methods may not be correct. As a result, the pre-stressed duct may not be densely filled and may exhibit stratification. Consequently, the liquid exuded from the cement may promote corrosion of the steel strand, and voids in the pre-stressed duct may lead to a reduction in the service performance and durability of the structure. The safety risk increases significantly because of these types of damage and fracture of pre-stressed ducts during service [2,3]. Therefore, there are two main aspects that must be considered during duct service: The grouting quality of the pre-stressed duct and the state of the steel strand in the duct.

The current research on pre-stressed ducts mainly focuses on the detection of the concrete filling, such as the detection of voids in grouted tendon ducts by the impact-echo method [4,5], and evaluation of the electromagnetic properties of an exuded cement product using capacitive sensors [6,7].

In our previous work on grouting quality evaluation, a proximity capacitive sensor was designed and used to evaluate the grouting quality of the duct. Detection principle is derived from industrial processing tomography (IPT) technique [8]. A new method based on the Q-factor was presented to identify the boundary position of the three-phase layer [9]. Previous studies used simplified conditions, and the presence of steel strands was not considered. However, state detection of steel strands is another important factor for structure safety evaluation.

For the damage detection of steel bars, Moustafa et al. used fractal analysis of guided ultrasonic waves to monitor the corrosion of post-tensioned concrete [10]. Sun et al. re-analyzed the principle of the magnetic flux leakage (MFL) technique, and the ‘escape’ phenomenon was explained from a physical point of view [11]. Tan et al. presented a fiber Bragg grating method to monitor rebar corrosion, and the corrosion could be detected by observing the Bragg wavelength shift [12]. Deng et al. analyzed the multi-source effect of a magnetization-based eddy current testing (MB-ECT) method using an equivalent source method, and the influence of the magnetizing current and the probe lift-off were investigated by finite element analysis (FEA). The experimental results revealed a multi-source effect in the mechanism of the MB-ECT sensors and provided the basic theory for precision crack evaluation [13]. Liu et al. designed a tunnel magnetoresistive-based MFL sensor array to detect the flaws of steel wire rope. However, the cement layer was not considered properly [14]. Liu and Xiao et al. designed a new type of sensor to measure the biased pulse magnetic response in a large-diameter steel stay cable. A finite element simulation was conducted to optimize the implementation plan of the new sensor, a prototype of the proposed sensor and a biased pulse current supply were developed, and the experimental results suggested the effectiveness of the new sensor for the assessment of surface and internal flaws [15]. The current research on steel bars in external post-tensioned pre-stressed ducts mainly focuses on the cracks and corrosion of steel bars, and scholars worldwide have achieved valuable results [16,17,18,19,20]. However, the steel strand distribution is also important, and little research has been conducted on this aspect.

The cross-sectional distribution of the steel bars is significant because it influences the load capacity of the duct and the bearing capacities and crack resistance of the structural components. According to the Chinese National Standard GB50204-2015, the positional deviation of pre-stressed tendons should be less than ±5 mm for structural components smaller than 300 mm [21]. Furthermore, the Chinese National Standard GB/T 30827-2014 requires that the steel strands should be as close to the center of the duct as possible [22]. However, in the real construction of pre-stressed tendons, the steel bars are not always placed in the designed positions. The buoyancy of the concrete during construction causes the pre-stressed tendons to deviate from the original design position. This deviation causes the structural components to be subjected to additional radial forces. Based on statistics of the bridges, where box girder cracking occurs, the positioning deviation of the steel bars can reach 20 to 30 mm [23]. Under the action of additional radial forces, when the steel bar floats by 20 mm, the maximum vertical stress on the box girder cross-section reaches 1.0 MPa; when the steel bar floats by 30 mm, the maximum vertical stress on the box girder cross-section reaches 1.3 MPa. Therefore, the positioning error of the pre-stressed tendons will greatly increase the local vertical tensile stress of the box girder. Eventually, this leads to cracking of the weak section of the box girder and endangers the safety of the bridge.

Locating the steel bars not only contributes to the assessment of the quality of the construction, but it also helps to prevent bridge accidents during service. In this paper, a capacitive sensing technique-based method is proposed to locate the cross-sectional distribution of a steel bar and provide useful information for follow-up work on the damaged locations of the steel bars. In Section 2, the working principle of the capacitive sensing technology and the detection methodology are briefly introduced and explained. Models with different positions of the steel strands in external post-tensioned pre-stressed ducts were constructed. Simulations and experiments are presented in Section 3, and the results are compared and analyzed simultaneously. A new method is proposed to improve the detection results of the experiment stage. The conclusions are given in Section 4.

## 2. Working Principle and Methodology

### 2.1. Working Principle of Capacitive Sensor

**Figure 2:**
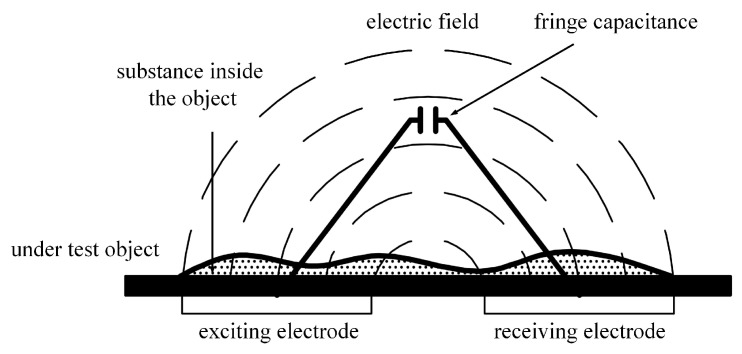
The working principle of the coplanar capacitive sensor.

A typical capacitive sensor is made of two traces: The exciting electrode and the receiving electrode. The working principle of the coplanar capacitive sensor is called the fringe effect of capacitance [24]. The electric field is formed between the two traces with an AC (Alternating current) excitation. When an unknown substance is placed inside the sensing area, the electric field and the permittivity between the two electrodes will change according to the material characteristics of the substance. By measuring the capacitance of the sensor, the existence and properties of the substance can be identified [25,26]. A diagram of the working principle is shown in Figure 2.

**Figure 3:**
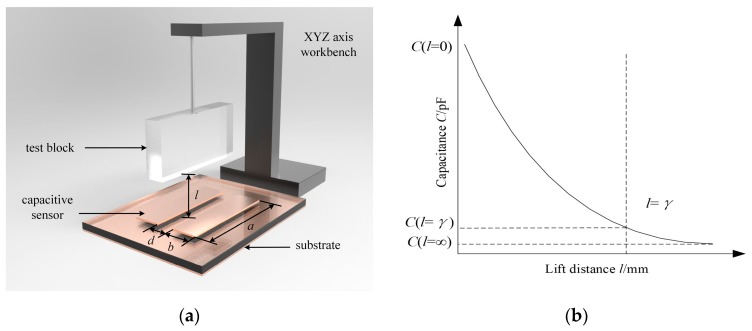
Penetration depth of the coplanar capacitive sensor: (**a**) Setup of the experiment; (**b**) the trend of the capacitance with different lift distances.

The penetration depth of the capacitive sensor indicates the sensing range of the sensor. The greater the penetration depth is, the larger the sensing range becomes. In our experiment, the test block (made of high-density polyethylene (HDPE)) was placed in the sensing area above the capacitive sensor, and the fringing capacitance of the capacitive sensor was measured with the test block at different lift distances, as shown in Figure 3a. In Figure 3a, *a*, *b*, and *d* are the width, length, and interval of the traces, respectively. The vertical distance between the test block and the surface of the capacitive sensor is the lift distance, *l*, and the effective penetration depth of the capacitive sensor is *γ*. The difference between the capacitance value, C (*l* = *γ*), at the position of the effective penetration depth and the minimum capacitance value, C (*l* = ∞), is equal to 3% of the difference between the maximum capacitance value, C (*l* = 0), and the minimum capacitance value, C (*l* = ∞), as shown in Equation (1) [27]:

(1) C(l=γ)−C(l=∞)=(C(l=0)−C(l=∞))⋅3%,

where *l* = 0 indicates that the test block is in full contact with the upper surface of the capacitive sensor. Furthermore, *l* = ∞ indicates that the test block is far from the upper surface of the capacitive sensor. In this situation, the change in *l* does not affect the capacitance between the electrodes. The trend of the capacitance with different lift distances is shown in Figure 3b.

### 2.2. Methodology

**Figure 4:**
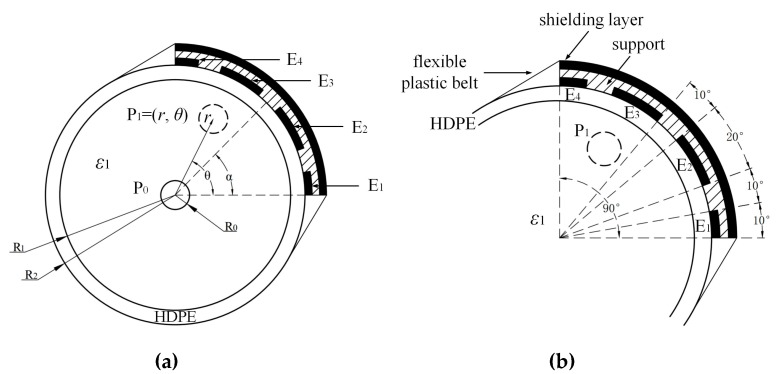
Capacitive sensor for the detection of the steel strand cross-section’s distribution. (**a**) Sensor placed on the external surface of duct and (**b**) sensor structure with 1/2 duct.

The cross-section of the sensor and pre-stressed duct is shown in Figure 4a, and the capacitive sensor structure consisted of four electrodes, named E1, E2, E3, and E4. Electrodes E1 to E4 were placed against on the external surface of the duct. The widths of electrodes E1, E2, E3, and E4 corresponded to the different central angles of 10°, 20°, 20°, and 10°, as shown in Figure 4b. The intervals between the proximity electrodes all corresponded to 10°. One quarter of the circumference was covered by the sensor. Therefore, the center of the sensor was at 45°. With the support of a flexible plastic belt, the traces could be easily fixed on the tube and rotated around the pipe. The shielding layer was placed on the outermost layer to resist the outside interference.

Polar coordinates were used to represent the positions of the steel bar and sensor. The coordinate system origin was the center of the duct. The angles were measured from the horizontal direction and rotated in an anti-clockwise direction. Three parameters, ***α***, ***θ***, and ***r***, were defined for the following simulations and experiments, as shown in Figure 4a. ***α*** is the angle position of the sensor, i.e., the angle between the horizontal direction and the line joining the center of the duct and the midpoint of the sensor. ***θ*** refers to the angle position of the steel bar, i.e., the angle between the horizontal direction and the line joining the center of the duct and the center of the steel bar. ***r*** is the distance between the center of the duct and the center of the steel bar. To complete the positioning, the four electrodes were divided into two pairs, E1, E4 and E2, E3. The sensor rotated around the pipe for scanning purposes. At the first stage, E1 was set as an excitation plate and E4 was a receiver. The capacitance between E1 and E4 were measured with rotation intervals of 45° (*α* = 0°, 45°, 90°,…, 360°) to locate the angle (*θ*) position of the steel strand. In the second round of measurements (second stage), E2 was set as an excitation plate, while E3 was a receiving electrode after *θ* was located. The capacitance between the two traces was measured to determine the center distance (*r*) of the steel strand.

To make the comparison between the simulated and measured values more intuitive, normalization was necessary, defined as follows:

(2) $$Nor_{ij}=\;\frac{C-C_{\min}}{C_{\max}\;-C_{\min}}$$

where *Nor_ij_* is the normalized simulated value between electrode Ei and Ej, *C* is the simulated or measured value, *C*_min_ is the minimum of simulated or measured value, and *C*_max_ is the maximum of the simulated or measured value.

A single capacitance value cannot be used to determine *r*. When *θ* was determined by the first round of measurements, the capacitance between E2 and E3 was measured at *θ* (*C*_1_). In addition, the capacitance at *θ* + 180° (*C*_2_) should be measured to provide a reference of the capacitance proportion. In this way, the *r* position of the steel strand could be calculated by the capacitance proportion, *P_c_*, as follows:

(3) $$P_{C}=\;C_{1}/(C_{1}\;+\;C_{2})$$

## 3. Simulations and Experiments

### 3.1. Phantom and Setup Conditions

Simulations are needed for the sensor structure design and verification of the proposed two-round measurement. The simulations were a crucial component of the research discussed below. 

**Figure 5:**
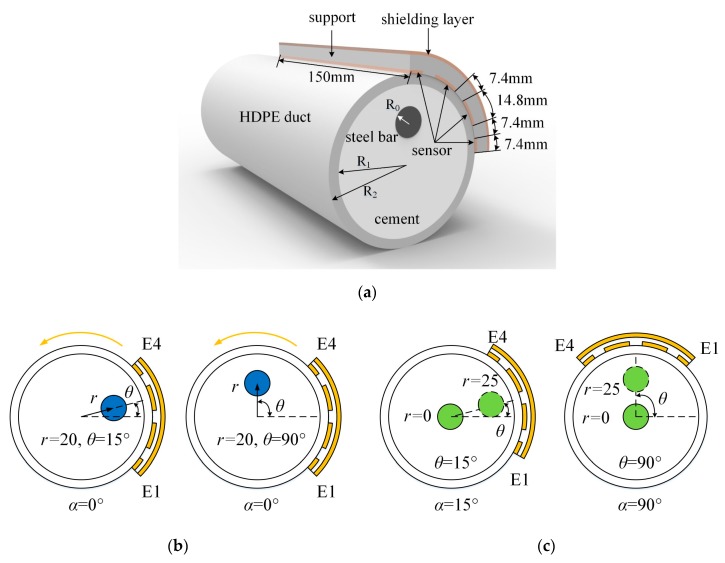
The phantoms for the simulations and experiments. (**a**) Schematic diagram of simulations and experiments; (**b**) the phantoms for *θ* detection in the first-round measurements; (**c**) the phantoms for *r* detection in the second-round measurements.

The simulations were performed using COMSOL Multiphysics, and the relevant parameters of the simulation were set as follows. Three-dimensional (3D) models were used for all the simulations. The dimensions, materials, and the relative permittivity values of the pre-stressed ducts, steel strand, and sensor in the experiment were the same as those in the simulations. The internal radius, R_1_, and external radius, R_2_, of the duct were 37.5 and 42.5 mm, respectively. A single steel bar was placed in the duct, which was filled with cement (*ε*_cement_ = 4). The radius, R_0_, of the steel bar was 7.5 mm. The widths of the electrodes were 7.4 mm for E1 and E4 and 14.8 mm for E2 and E3. The lengths and intervals of the electrodes were 150 and 7.4 mm, respectively, for all traces. The ducts and the support for the sensor were made of HDPE, and the entire capacitive sensor and shielding layer were made of copper foil. A schematic diagram of the simulations and experiments is shown in Figure 5a. The cement for grouting was standard Portland cement, and the water:cement ratio was 1:0.65 [28].

In the simulation stage, the permittivity of cement, HDPE, and water were set to *ε*_cement_ = 4, *ε*_HDPE_ = 2.3, and *ε*_warer_ = 80, respectively. The permittivity depends on various experimental parameters, such as the cement preparation process (water and cement) and chemical reaction process (steel and water). The AC excitation was set to 5 V for the simulations and experiments. The excitation frequency for the experiment was 1 MHz. However, this could not be set in the simulation. The steel bar and shielding layer were grounded in the simulations and experiments. 

The location process can be divided into two stages. As mentioned in Section 2, the sensor scanning interval was 45° in the first round of measurements, i.e., α = 0°, 45°, 90°, … Two positions of the steel bar were considered. *θ* was set to 15° and 90° with the same *r =* 20 mm. Therefore, the position of the steel strand can be represented as *P*(*r*, *θ*), as shown in Figure 5b. In the second stage, for the detection of *r*, due to its symmetrical structure, two sets of models were set, as shown in Figure 5a: (1) *θ* = 15°, *α* = 15°, where *r* varied from the center of the duct to 25 mm with intervals of 5 mm, and (2) *θ* = 90°, *α* = 90°, where *r* varied from the center of the duct to 25 mm with intervals of 5 mm. The sensor must also be placed at *θ* + 180° to further identify the exact segment containing the steel strand. The phantoms for the simulations and experiments are shown in Figure 5.

**Figure 6:**
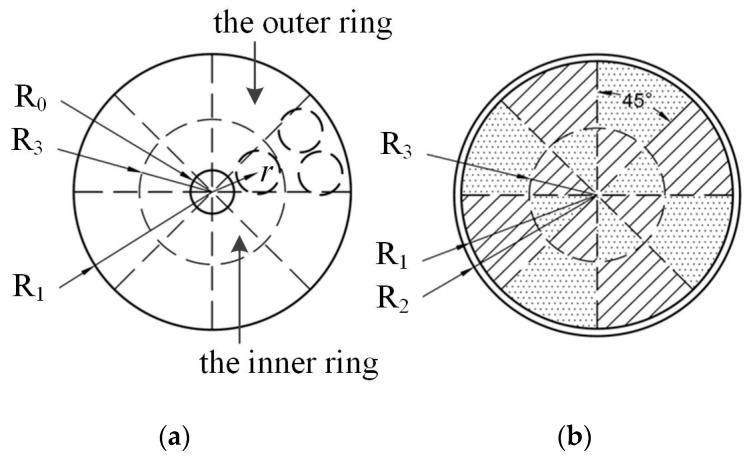
Cross-sectional area division of the duct model. (**a**) Placement of the steel bar in the duct; (**b**) the 16 segments.

Since the diameter of the steel bar was 15 mm in this study, and the inner radius of the duct was 37.5 mm, we divided the entire cross-sectional area into 16 segments with 45° for each section, as shown in Figure 6. Such segmentation is sufficient for engineering applications. Note that the internal circle’s radius, R_3_, was set to 22.5 mm (corresponding to the steel bar at *r* = 15 mm). Thus, we only needed to identify in which area the steel strand is located instead of determining its position accurately.

### 3.2. Simulation Results and Analysis

#### 3.2.1. A. Detection of the Angle Position, θ, of the Steel Strand

**Figure 7:**
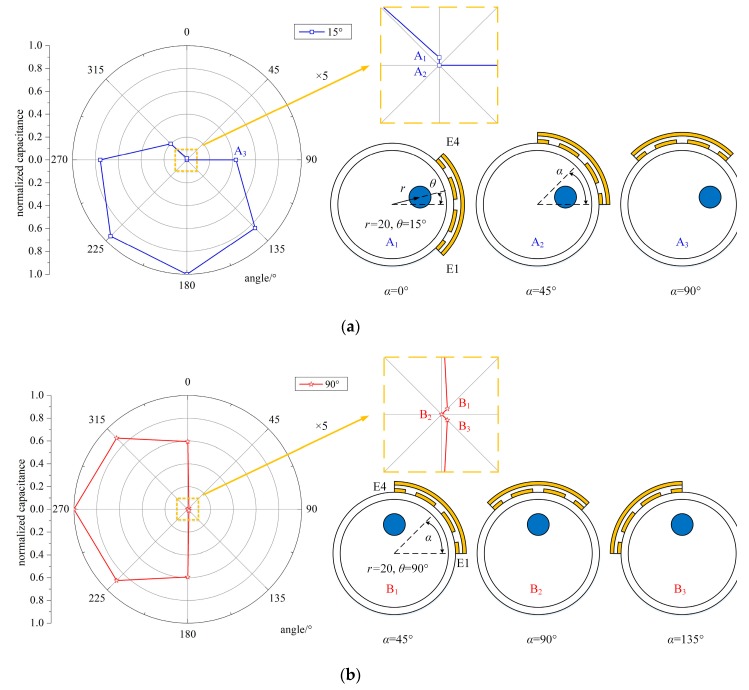
The normalized simulated capacitance between E1 and E4 and the special positions for *θ* detection. (**a**) *θ* = 15°, *r* = 20 mm, *α* = 0°, 45°, 90°; (**b**) θ = 90°, *r* = 20 mm, α = 45°, 90°, 135°.

The capacitance between E1 and E4 was obtained by the simulations, and the normalized simulated values were calculated using Equation (2). The simulated capacitance and normalized values between E1 and E4 are presented in Table 1 and Figure 7. The special positions for *θ* detection are also presented, and part of the area containing special points is magnified five times in Figure 7.

For *θ* = 15° and *r* = 20 mm, three characteristic points were selected, named A_1_, A_2_, and A_3_. A_1_, A_2_, and A_3_ corresponded to the normalized capacitance value, *C*_n_^14^, when the position of the sensor, *α*, was 0°, 45°, and 90°, respectively. We can derive from Figure 7a that (i) A_2_ was the lowest point among the normalized values. If the steel bar was in the sensing area of the capacitive sensor, the capacitance between the two electrodes would decrease. Therefore, the steel bar was in the area of the sensor electrode at 45°, which was in the 0° to 90° range. (ii) Note that A_1_ was basically equal to 0, as was A_2_, which was different from the other points. Thus, the steel strand was also in the area of the sensor electrode at 0°, corresponding to the −45° to 45° range. (iii) Based on the above analysis, the steel bar was placed in the 0° to 45° segment, which was the overlapped range.

For *θ* = 90° and *r* = 20 mm, three characteristic points were marked, named B_1_, B_2_, and B_3_. B_1_, B_2_, and B_3_ corresponded to the normalized capacitance value, *C*_n_^14^, when the position of the sensor, *α*, was 45°, 90°, and 135°, respectively. We can derive from Figure 7b that (i) B_2_ was the lowest point among the normalized simulated values. If the steel bar was in the sensing area of the capacitive sensor, the capacitance between the two electrodes would decrease. Thus, the steel bar was in the area of the sensor electrode at 90°, which was in the 45° to 135° range. (ii) Note that B_1_ and B_3_ were basically equal to 0, as was B_2_, which meant that the steel bar was also in the area of the sensor electrode at 45° and 135°, corresponding to the 0° to 90° and 90° to 180° ranges, respectively. (iii) Combining the information above, the steel bar was placed in the 45° to 135° segment. In addition, B_1_ was equal to B_3_, which meant that the steel bar was in a symmetric position when the sensor electrode was at 45° and 135°. Therefore, the steel bar was in the middle of the 45° to 135° range, and its position angle was 90°.

By the method above, we located the steel bar at least in one 45° segment, and for the special angles (90°), the proposed method can even determine the exact position of the steel bar.

#### 3.2.2. B. Detection of the Center Distance, r, of the Steel Strand

**Figure 8:**
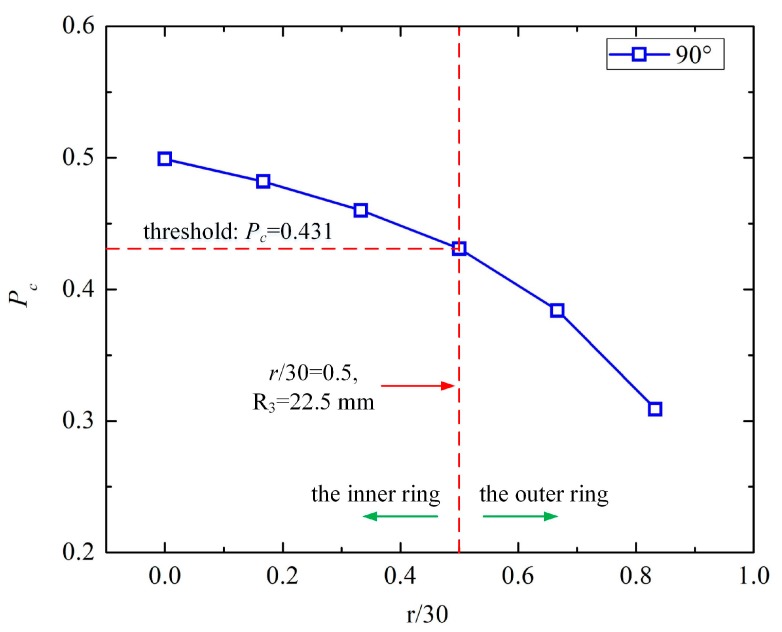
The trend of the relative proportion of capacitance and center distance percentage.

The capacitance between E2 and E3 (*C*_1_^23^) was acquired by simulations. The capacitance at *θ* + 180° (*C*_2_^23^) was also simulated to obtain the area of the steel bar. To compare the capacitance trend more conveniently, the relative proportion of capacitance was calculated using Equation (3), as demonstrated in Table 2 and Figure 8. When the angle of the sensor was fixed at the angle of the steel bar, the sensor and duct had symmetrical structures. Thus, there was no difference between *θ* = 15° and *θ* = 90°. We only plotted the relative proportion of the capacitance for one phantom in Figure 8. Accordingly, for *θ* = 90 and *α* = 90°, *r* ranged from 0 to 25 mm with increments of 5 mm.

From Table 2 and Figure 8, some conclusions can be derived: 

(i) As the center distance, *r*, of the steel bar increased, the capacitance between E2 and E3 (***C*_1_^23^**) decreased rapidly and the capacitance at *θ* + 180° (***C*_1_^23^**) increased gradually, which contributed to the fast decline of ***C*_1_^23^**/(***C*_1_^2^** + ***C*_2_^23^**). 

(ii) When the relative position between the sensor and steel bar was fixed, it was symmetrical for every *θ* in the one-phase model. Thus, the two sets of simulations shared the same trend. Therefore, we set *P_c_* = ***C*_1_^23^**/(***C*_1_^23^** + ***C*_2_^23^**) = 0.431 as the threshold of *r* = 15 mm (*r*/30 = 0.5, R_3_ = 22.5 mm). 

We identified the steel bar location as either in the inner circle (0 mm ≤ *r* ≤ 15 mm) or the outer ring (15 mm ≤ *r* ≤ 30 mm). With the two rounds of measurements, the steel bar could be located in 1 of 16 segments. The improved sensor structure could locate the steel bar positions based on the simulated results.

### 3.3. Experimental Results and Analysis

#### 3.3.1. A. Detection of the Angular Position, θ, of the Steel Strand

**Figure 9:**
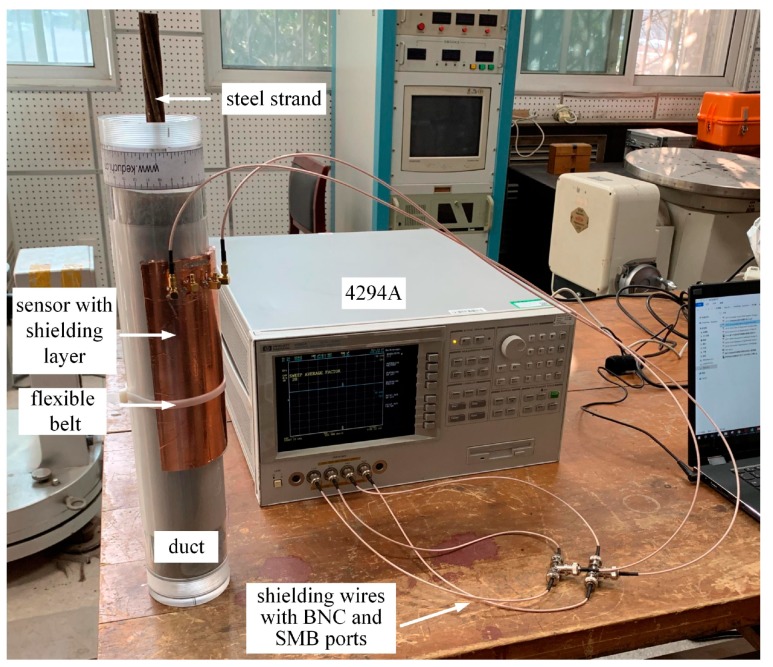
Diagram of the experimental device.

The parameters of the experiments were identical to the simulations. The capacitance was measured using an impedance analyzer (Agilent 4294A, Beijing, China), and a diagram of the experimental device is shown in Figure 9.

In the first round of measurement for the angular position, the experimental capacitance values between E1 and E4 were negative, as shown in Table 3, which were invalid measurements. It is the stray capacitance that influenced the experimental results. When the steel bar appeared in the sector that was covered by the sensing area of the capacitive sensor, four capacitors were formed. For example, when E1 was used for excitation, the original capacitance, *C*, was formed by E1 and E4. The stray capacitance, *C_s_*_1_, *C_s_*_2_, and *C_s_*_3_, were formed by E1 and the steel bar, E1 and the shielding layer, and E1 and the cement, respectively. As a result, the measured capacitance between E1 and E4 dropped below zero. Therefore, the quality factor (Q-factor) was introduced for further experiments of the angular position’s identification.

In our previous research, the boundary detecting method for post-tensioned pre-stressed ducts based on Q-factor analysis was introduced, which could effectively identify the boundary position of the three-phase duct model. Generally, *Q* is calculated for a capacitor as follows:

(4) $$Q=\;\frac{X_{C}}{R_{C}}\;=\;\frac{1}{\omega_{0}CR_{C}}$$

where *ω*_0_ is the resonance frequency (radians per second), *C* is the capacitance, *X_C_* is the capacitive reactance, and *R_C_* is the series resistance of the capacitor.

The frequency sweep was performed using an Agilent 4294A. The frequency range was from 40 Hz to 100 MHz. The frequency sweep was conducted for each *α* (0°, 45°, 90°…). The maximum value of the Q-factor was defined as MAX, and the corresponding frequency was ***f*_max_**_._ Since MAX and *f*_max_ were unstable within the frequency range, trace bandwidth analysis was performed. The cutoff point was obtained by dividing the MAX by 2. The cutoff points were searched for toward both sides of the measurement parameter axis, using the current position of the MAX as the center. The bandwidth (distance between the two cutoff points), center value (midpoint of the two cutoff points), and corresponding frequency, ***f*_c_**, were obtained. The steps for calculating the center frequency, *f_c_*, were introduced in detail in our previous work [9].

**Figure 10:**
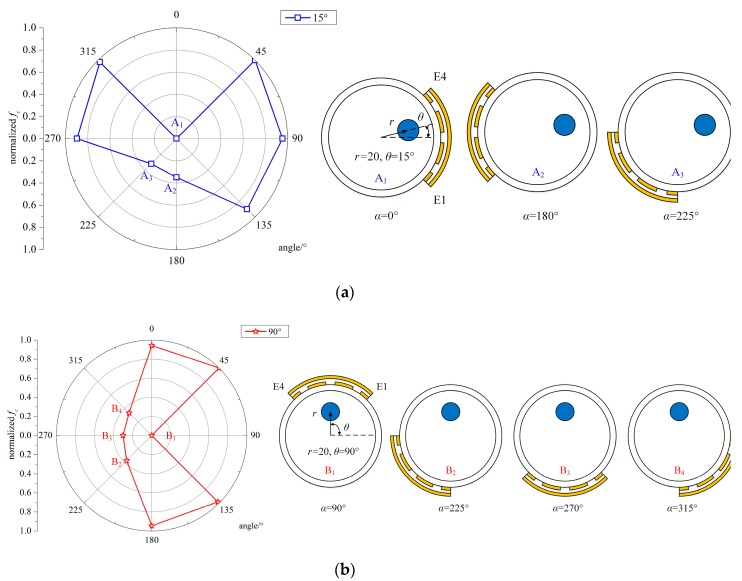
The normalized simulated capacitance between E1 and E4 and the special positions for *θ* detection. (**a**) *θ* = 15°, *r* = 20 mm, *α* = 0°, 180°, 225°; (**b**) θ = 90°, *r* = 20 mm, α = 90°, 225°, 270°, 315°.

The center frequency, *f_c_*, was selected for angle position detection, and the measured data are listed in Table 4. Normalization was conducted for better identification of the steel strand angle position. The sensor positions for *θ* = 15°, *r* = 20 mm and *θ* = 90°, *r* = 20 mm are also shown in Figure 10.

For *θ* = 15° and *r* = 20 mm, three characteristic points were marked, named A_1_, A_2_, and A_3_. A_1_, A_2_, and A_3_ corresponded to the normalized capacitance value, *C*_n_^14^, when the position of the sensor, *α*, was 0°, 180°, and 225°, respectively. (i) A_1_ was the lowest point among the normalized values. Therefore, the steel bar was in the area of the sensor electrode at 0°, which was in the −45° to 45° range. (ii) A_2_ and A_3_ were the lowest points other than A_1_, and their normalized values were around 0.3, which means that the steel strand was in the opposite area of *α* = 180° and *α* = 225°, corresponding to the −45° to 90° range, respectively. (iii) Note that A_2_ was approximately equal to A_3_, so the steel bar was in the middle region of −45° to 90°, that is 0° to 45°. Therefore, the steel bar could be located in the 0° to 45° segment.

For *θ* = 90° and *r* = 20 mm, four characteristic points were marked, named B_1_, B_2_, B_3_ and B_4_. B_1_, B_2_, B_3_, and B_4_ corresponded to the normalized capacitance value, *C*_n_^14^, when the position of the sensor, *α*, was 90°, 225°, 270°, and 315°, respectively. (i) B_1_ was the lowest point among the normalized values. Thus, the steel bar was in the area of the sensor electrode at 90°, which was in the 45° to 135° range. (ii) B_2_, B_3_, and B_4_ were the lowest points other than B_1_, and their normalized values were around 0.3, which means the steel strand was in the opposite area of *α* = 225°, 270°, 315°, i.e., the range from 0° to 180°. (iii) It is remarkable that the steel bar was in the symmetrical position when the sensor electrode was at 45° and 135°, 0° and 180°, and 225° and 315°, since their values were approximately the same. Therefore, the steel bar was in the middle of the 45° to 135° range, and its position angle was 90°.

By the method above, we could locate the steel bar at least in one 45° segment, and for the special angles (90°), the proposed method could even determine the exact position of the steel bar. Furthermore, the accuracy of the positioning could be improved by reducing the scanning interval of the sensor. For instance, we could set α = 30° or 15°, and the steel bar could be located in one segment of 30° or 15°.

#### 3.3.2. B. Detection of the Center Distance, r, of the Steel Strand

**Figure 11:**
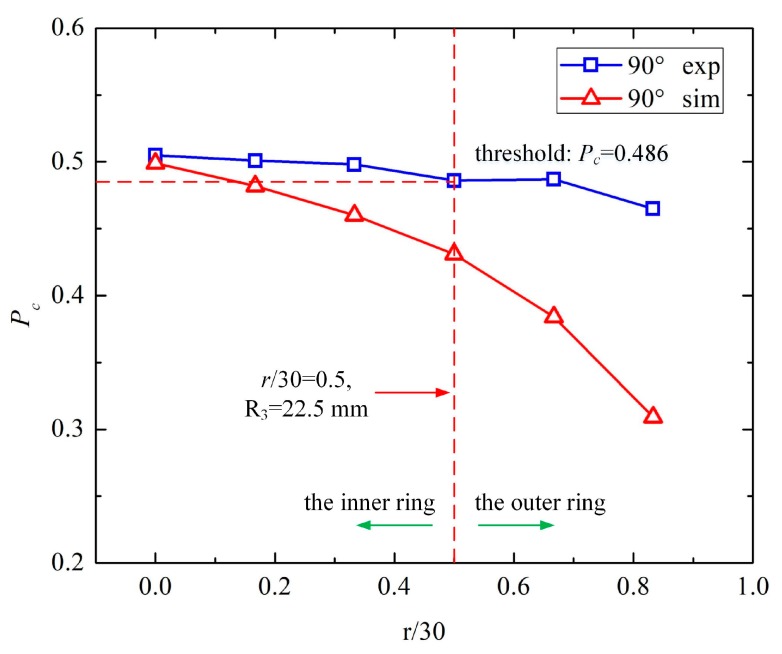
The measured and simulated relative proportion of the capacitance between E2 and E3 (*P_c_*).

**Figure 12:**
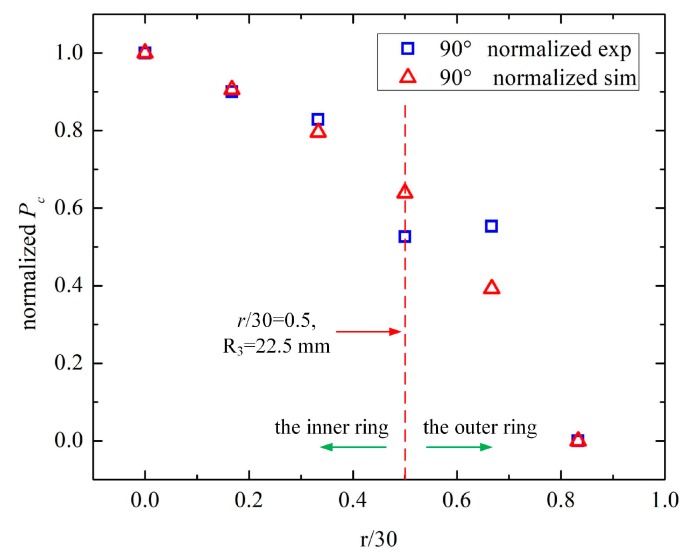
The normalized *P_c_* of the experiment and simulation.

In the second-round of measurements for the center distance, only one configuration was studied: *θ* = 90° and *α* = 90°, where *r* ranged from 0 to 25 mm with increments of 5 mm, due to its symmetrical structure. The capacitance between E2 and E3 (***C*_1_^23^**), and the capacitance of *θ* + 180° (***C*_2_^23^**) were measured using an Agilent 4294A, as described above. The excitation frequency was 1 MHz. The measured capacitance and relative proportion of the capacitance between E2 and E3 (*P_c_*) are demonstrated in Table 5 and Figure 11. In addition, normalization was conducted for a better comparison between the simulation and measurement, as illustrated in Table 6 and Figure 12.

From Table 5 and Table 6 and Figure 11 and Figure 12, some conclusions can be drawn: 

(i) As the center distance, *r*, of the steel bar increases, the capacitance between E2 and E3 (*C*_1_^23^) decreased gradually, and the capacitance of *θ* + 180° (*C*_2_^23^) increased slowly, which contributed to the slight decrease in *C*_1_^23^/(*C*_1_^23^ + *C*_2_^23^). 

(ii) In Figure 11, the experimental and simulated results (*P_c_*) followed the same decreasing trend as *r* increased, indicating the validity of the experiments. The measured *P_c_* decreased more slowly than the simulated one because the stray capacitance weakened the influence of the center distance, *r*, on *P_c_*. 

(iii) For Figure 12, the normalized *P_c_* of the experiments and simulations shared the same decreasing tendency. There were gaps between the three measured values and the simulation, that is *r*/30 = 0.333, 0.5, and 0.667. On the one hand, due to the small variation of *P_c_* in the measurements, the normalization amplified the variable quantity. On the other hand, the position error of the steel bar caused by the twisting of the steel strand and the setting of the cement contributed to the differences between the experiments and simulations.

Therefore, we set *P_c_* = *C*_1_^23^/(*C*_1_^23^ + *C*_2_^23^) = 0.486 as the threshold of *r* = 15 mm (*r*/30 = 0.5, R_3_ = 22.5 mm) to determine whether the steel bar was located in the inner circle (0 mm ≤ *r* ≤ 15 mm) or the outer ring (15 mm ≤ *r* ≤ 30 mm). With the two rounds of measurements, the steel bar could be located in 1 of 16 segments. In the first-round of measurements, its angle (*θ*) was inspected in the 45° range. In the second round of measurements, its center distance (*r*) could be identified in one of the two rings. Thus, the improved sensor structure could locate the steel bar positions.

## 4. Conclusions

In this research project, the position of a single steel strand in a grouting duct was successfully identified by simulations and experiments based on capacitive sensing technology. From the simulated and experimental results, we reached the following conclusions:

(1) The proposed sensor structure with four electrodes demonstrated the effectiveness of detecting the distribution of a single steel bar in an external post-tensioned pre-stressed duct using simulations and experiments. Two measurement steps were presented for the detection of the angular position and center distance of the steel strand.

(2) For the detection of the angular position, *θ*, of the steel bar, the capacitance between E1 and E4 was measured, and it was effective for the simulations. However, it was invalid for practical experiments. A method based on the Q-factor was presented, and it successfully located the steel strand position in the 45° area.

(3) For the detection of the center distance, *r*, of the steel bar, the capacitance between E2 and E4 was measured, and it was valid for the simulations. The detection differences between the simulations and experiments at center distance, *r*, were analyzed, and the results were acceptable. After two steps of measurements in the simulations and experiments, the steel cross-section distribution could be identified in 1 of 16 segments.

(4) Future research shall place an emphasis on: (a) How the stray capacitance quantitatively affects the experimental results; (b) determining the dependence of the Q-factor on the steel and center frequency; and (c) sensor structure optimization.

## Figures and Tables

**Table 1:** The simulated capacitance (pF) and normalized values between E1 and E4.

α/°	*Θ* = 15°, *r* = 20 mm		*Θ* = 90°, *r* = 20 mm	
	*C*_14_*/*pF	Normalized *C_n_*^14^	*C*_14_*/*pF	Normalized *C_n_*^14^
0°	0.015	0.014	0.057	0.594
45°	0.014	0.000	0.017	0.014
90°	0.044	0.429	0.016	0.000
135°	0.073	0.843	0.017	0.014
180°	0.084	1.000	0.057	0.594
225°	0.080	0.943	0.077	0.884
270°	0.067	0.757	0.085	1.000
315°	0.028	0.200	0.077	0.884
360°	0.015	0.014	0.057	0.594

**Table 2:** The simulated capacitance (pF) and relative proportion of capacitance between E2 and E3.

r/mm	r/30	15°(*C*_1_^23^)	15° + 180°(*C*_2_^23^)	15° *P_c_*	90°(*C*_1_^23^)	90° + 180°(*C*_2_^23^)	90° *P_c_*
0	0.000	1.218	1.212	0.501	1.205	1.209	0.499
5	0.167	1.168	1.246	0.484	1.155	1.244	0.482
10	0.333	1.095	1.272	0.463	1.083	1.269	0.460
15	0.500	0.986	1.291	0.433	0.974	1.288	0.431
20	0.667	0.823	1.306	0.386	0.812	1.303	0.384
25	0.833	0.599	1.319	0.312	0.589	1.316	0.309

**Table 3:** The measured capacitance (fF) and normalized values between E1 and E4.

α/°	*θ* = 15°, *r* = 20 mm		*θ* = 90°, *r* = 20 mm	
	*C*_14_/pF	Normalized *C_n_*^14^	*C*_14_/pF	Normalized *C_n_*^14^
0°	−19	1.000	−28	0.583
45°	−19	1.000	−20	0.917
90°	−27	0.652	−18	1.000
135°	−37	0.217	−20	0.917
180°	−42	0.000	−30	0.500
225°	−40	0.087	−38	0.167
270°	−31	0.478	−42	0.000
315°	−21	0.913	−37	0.208
360°	−19	1.000	−28	0.583

**Table 4:** The measured *f_c_* (MHz) and normalized values between E1 and E4.

α/°	*θ* = 15°, *r* = 20 mm		*θ* = 90°, *r* = 20 mm	
	*f_c_*/MHz	Normalized *f_c_*	*f_c_*/MHz	Normalized *f_c_*
0°	0.538	0.000	1.079	0.943
45°	1.126	1.000	1.112	1.000
90°	1.099	0.955	0.534	0.000
135°	1.066	0.898	1.102	0.982
180°	0.743	0.350	1.081	0.946
225°	0.726	0.320	0.748	0.370
270°	1.065	0.897	0.707	0.299
315°	1.112	0.976	0.727	0.334
360°	0.538	0.000	1.079	0.943

**Table 5:** The measured capacitance (pF) and relative proportion of capacitance between E2 and E3 (*P_c_*).

r/mm	r/30	90°(*C*_1_^23^)	90°+180°(*C*_2_^23^)	90° *P_c_*
0	0.000	0.258	0.253	0.505
5	0.167	0.273	0.272	0.501
10	0.333	0.255	0.257	0.498
15	0.500	0.243	0.257	0.486
20	0.667	0.245	0.258	0.487
25	0.833	0.239	0.275	0.465

**Table 6:** The normalized *P_c_* of the experiment and simulation.

r/mm	r/30	90° Experiment		90° Simulation	
		*P_c_*	Normalized *P_c_*	*P_c_*	Normalized *P_c_*
0	0.000	0.505	1.000	0.499	1.000
5	0.167	0.501	0.900	0.482	0.906
10	0.333	0.498	0.828	0.460	0.796
15	0.500	0.486	0.527	0.431	0.639
20	0.667	0.487	0.554	0.384	0.393
25	0.833	0.465	0.000	0.309	0.000
